# Supplementary material for: The causal role of circulating inflammatory markers in osteoporosis: a bidirectional Mendelian randomized study
Source: Front Immunol. 2024 Jul 18;15:1412298. doi: 10.3389/fimmu.2024.1412298 (PMC11291241; doi:10.3389/fimmu.2024.1412298)
Supplement: Supplementary file 1 [file DataSheet_1.zip › supplementary material/CIM-OSTEOPOROSIS /GCST90274842.csv_forest.pdf]

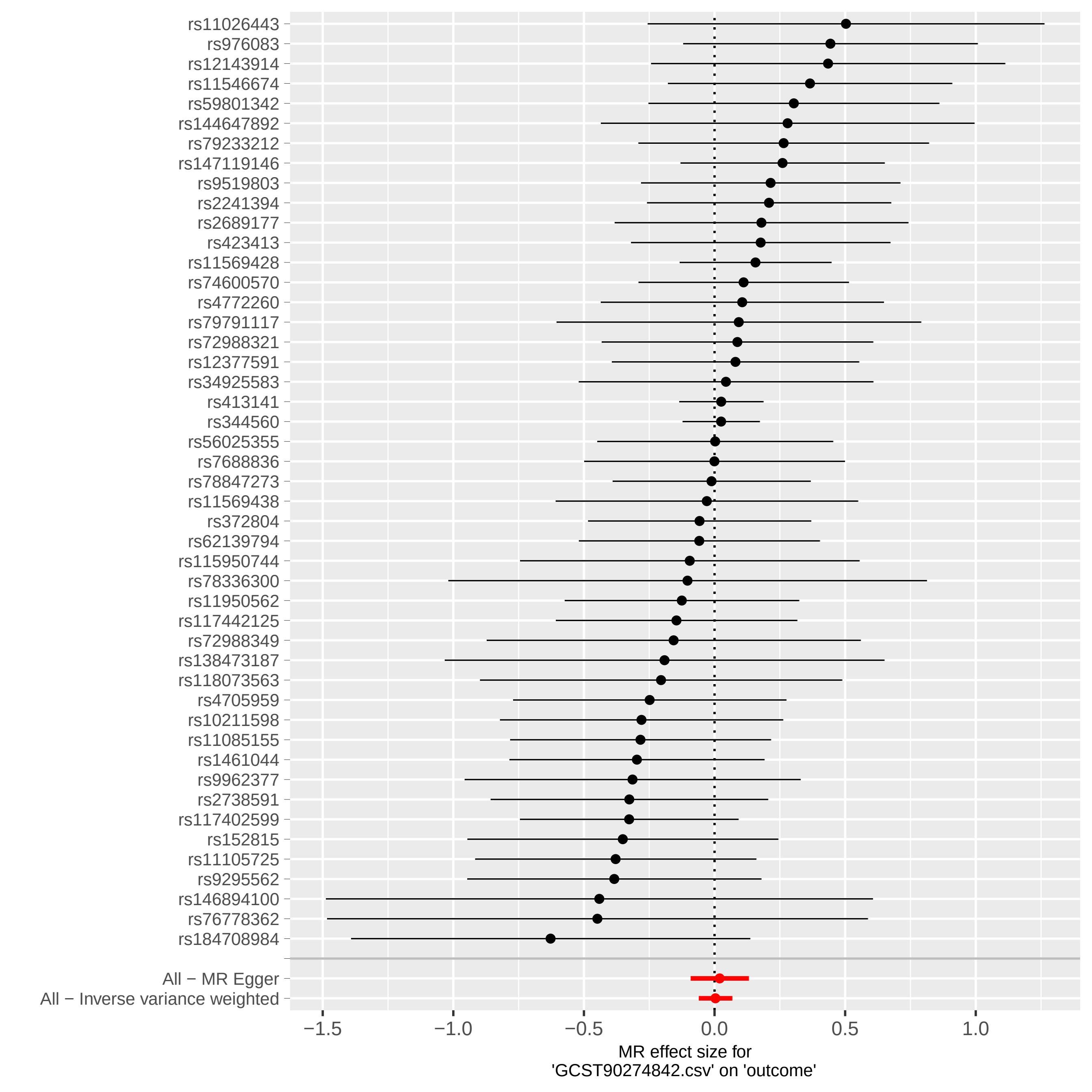

rs11026443
rs976083
rs12143914
rs11546674
rs59801342
rs144647892
rs79233212
rs147119146
rs9519803
rs2241394
rs2689177
rs423413
rs11569428
rs74600570
rs4772260
rs79791117
rs72988321
rs12377591
rs34925583
rs413141
rs344560
rs56025355
rs7688836
rs78847273
rs11569438
rs372804
rs62139794
rs115950744
rs78336300
rs11950562
rs117442125
rs72988349
rs138473187
rs118073563
rs4705959
rs10211598
rs11085155
rs1461044
rs9962377
rs2738591
rs117402599
rs152815
rs11105725
rs9295562
rs146894100
rs76778362
rs184708984
All − MR Egger
All − Inverse variance weighted
−1.5
−1.0
−0.5
0.0
0.5
1.0
MR effect size for
'GCST90274842.csv' on 'outcome'
